# A new in vitro monitoring system reveals a specific influence of *Arabidopsis* nitrogen nutrition on its susceptibility to *Alternaria brassicicola* at the seedling stage

**DOI:** 10.1186/s13007-022-00962-3

**Published:** 2022-12-08

**Authors:** Thibault Barrit, Claire Campion, Sophie Aligon, Julie Bourbeillon, David Rousseau, Elisabeth Planchet, Béatrice Teulat

**Affiliations:** Institut Agro, Univ Angers, INRAE, IRHS, SFR QUASAV, 49000 Angers, France

**Keywords:** Ammonium, Image analysis, Necrosis, Necrotrophic fungus, Nitrate, Root, Seedling, Symptom

## Abstract

**Background:**

Seedling growth is an early phase of plant development highly susceptible to environmental factors such as soil nitrogen (N) availability or presence of seed-borne pathogens. Whereas N plays a central role in plant-pathogen interactions, its role has never been studied during this early phase for the interaction between *Arabidopsis thaliana* and *Alternaria brassicicola*, a seed-transmitted necrotrophic fungus. The aim of the present work was to develop an in vitro monitoring system allowing to study the impact of the fungus on *A. thaliana* seedling growth, while modulating N nutrition.

**Results:**

The developed system consists of square plates placed vertically and filled with nutrient agar medium allowing modulation of N conditions. Seeds are inoculated after sowing by depositing a droplet of conidial suspension. A specific semi-automated image analysis pipeline based on the Ilastik software was developed to quantify the impact of the fungus on seedling aerial development, calculating an index accounting for every aspect of fungal impact, namely seedling death, necrosis and developmental delay. The system also permits to monitor root elongation. The interest of the system was then confirmed by characterising how N media composition [0.1 and 5 mM of nitrate (NO_3_^−^), 5 mM of ammonium (NH_4_^+^)] affects the impact of the fungus on three *A. thaliana* ecotypes. Seedling development was strongly and negatively affected by the fungus. However, seedlings grown with 5 mM NO_3_^−^ were less susceptible than those grown with NH_4_^+^ or 0.1 mM NO_3_^−^, which differed from what was observed with adult plants (rosette stage).

**Conclusions:**

The developed monitoring system allows accurate determination of seedling growth characteristics (both on aerial and root parts) and symptoms. Altogether, this system could be used to study the impact of plant nutrition on susceptibility of various genotypes to fungi at the seedling stage.

**Supplementary Information:**

The online version contains supplementary material available at 10.1186/s13007-022-00962-3.

## Background

Rapid and homogeneous seedling growth is crucial for successful crop establishment and yield. However, this early stage of plant development is highly susceptible to environmental conditions including variations of nitrogen (N) availability in the soil [1, 2], as well as development of diseases generally caused by seed- and soil-borne plant pathogens [3, 4].

Nitrogen plays a crucial role driving plant development, growth and yield. Nitrate (NO_3_^−^), the main form and source of N absorbed by seedlings and young plants in temperate regions, also acts as a major signal molecule modulating plant metabolism, growth and plant defence responses [5]. Nitrogen uptake and metabolism are also central to plant defence mechanisms as they provide the material for defence compounds and allow N mobilisation away from pathogens [6]. For instance, the phytoalexin camalexin, which has been shown to have an effect in the *Arabidopsis thaliana*—*Alternaria brassicicola* interaction [7], is derived from the tryptophan amino acid [8]. Amino acids, including methionine, phenylalanine and tryptophan, are also the basis of phytoanticipins such as glucosinolates in plants of the *Brassicaceae* family [9] which, once broken down into isothiocyanates and nitriles, may be toxic to fungi, bacteria and insects [10]. Another important molecule involved in plant defence mechanisms and derived from NO_3_^−^, is nitric oxide (NO). NO is rapidly accumulated during biotic stress conditions and plays a role in phytoalexin accumulation [11] and salicylic acid biosynthesis [12], a major pathway for plant resistance to infection leading for instance to hypersensitive response [13]. Nitrogen is not only vital for the plants, but also for the fungal pathogens, the latter being entirely dependent on plant N sources, such as NO_3_^−^, ammonium (NH_4_^+^) and free amino acids, for their growth [6]. This makes the host N metabolism a direct factor affecting fungal growth, by modulating the N sources available for the invader.

The role of N on pathogenicity is unclear. Indeed, a large supply of exogenous N improves plant nutrition and defences but can also promote disease development, by increasing the availability of N compounds assimilable by the pathogen [14, 15]. It has been shown that high N fertilisation could promote the disease [16, 17] or increase host resistance [18], which would suggest that the role of N in the plant-pathogen interaction is complex and pathosystem specific. In addition to the overall availability of N, its form also plays an important role in plant-pathogen interaction. Studies have shown that NH_4_^+^ nutrition could increase disease severity compared to NO_3_^−^ [19, 20], while the contrary was observed in other pathosystems [21], notably *A. thaliana—A. brassicicola* [22].

Until now, inorganic N fertilisers and conventional agrochemicals, such as fungicides, have been widely used for many years to protect seeds and seedlings against various stresses in the seedbed and to increase seedling establishment. However, the agro-ecological transition requires a substantial reduction of N input and seed treatment with fungicides. These practices would however promote the presence of seed-borne pathogens such as necrotrophic fungi on both seeds and seedlings. Moreover, N fertilisers do not only affect seedling growth but can also have different effects on disease development, shifting the balance in favour of the host or of the pathogen as reported at other stages of plant development [6, 16]. In this context and surprisingly, the impact of N fertilisation on the interaction between plants and pathogens has been little studied at the seedling stage and requires more investigation.

In order to study the impact of N nutrition on plant-pathogen interaction during seedling development, one would need a protocol allowing symptom severity, fungus growth and seedling development (i.e. both aerial and root parts) to be monitored, in a nutrient controlled sterile environment. Studies on early plant stage interaction with pathogens commonly involve an inoculation on seven to eighteen days old seedlings growing in soil [17, 23, 24] or in a Petri dish [25], neither permitting the study of the interaction during germination, early seedling growth nor root development. In order to investigate the interaction as early as possible, inoculation has to take place directly on the seeds, by placing them in contact with dry spores or by immersing them in an inoculum suspension [26–28]. The best option to achieve a sterile and controlled environment allowing to monitor both aerial and root growth seems to be the use of agar media [29]. A protocol combining a seed inoculation with a modular sterile agar medium could therefore be a good solution to study the role of N on the plant-pathogen interaction during seedling development.

In the present work, we developed a monitoring system focusing on the interaction between the model plant for the *Brassicacae* family *A. thaliana* and the necrotrophic fungus *A. brassicicola*. *Arabidopsis thaliana* is a practical and already well studied model for host–pathogen interactions [30], and the interaction with *A. brassicicola,* in particular, has been abundantly used as a model for diseases caused by fungal necrotrophs [31]. This fungus is responsible for the black spot disease on leaves of *Brassicaceae* plants [32], leading to important worldwide economic losses [33]. Infection with *A. brassicicola* is however not limited to the leaves, but this fungus can affect all aerial parts of the plant, such as siliques and seeds in particular. Such a wide infection spectrum constitutes an important mode of conservation and transmission of the fungal inoculum and therefore makes of *A. brassicicola* a major seed-borne disease leading to seedling damping-off [34–36]. The monitoring system, allowing both homogenised seed contamination and following seedling aerial and root development by image analysis, was firstly developed on one *A. thaliana* ecotype grown in one N nutrition medium, 5 mM NO_3_^−^. To validate its interest for screening the impact of the fungus on seedling growth, the protocol was then used to characterise the response to the fungus of three ecotypes with contrasting susceptibilities, on three N media varying in forms and concentration (5 mM NH_4_^+^, 0.1 mM NO_3_^−^ and 5 mM NO_3_^−^). Finally, the results obtained at seedling stage with this developed monitoring system were compared with behaviours at adult stage (*i.e.* rosette).

## Results

### The developed monitoring system allows studying how the fungus affects seedling growth

**Fig. 1:**
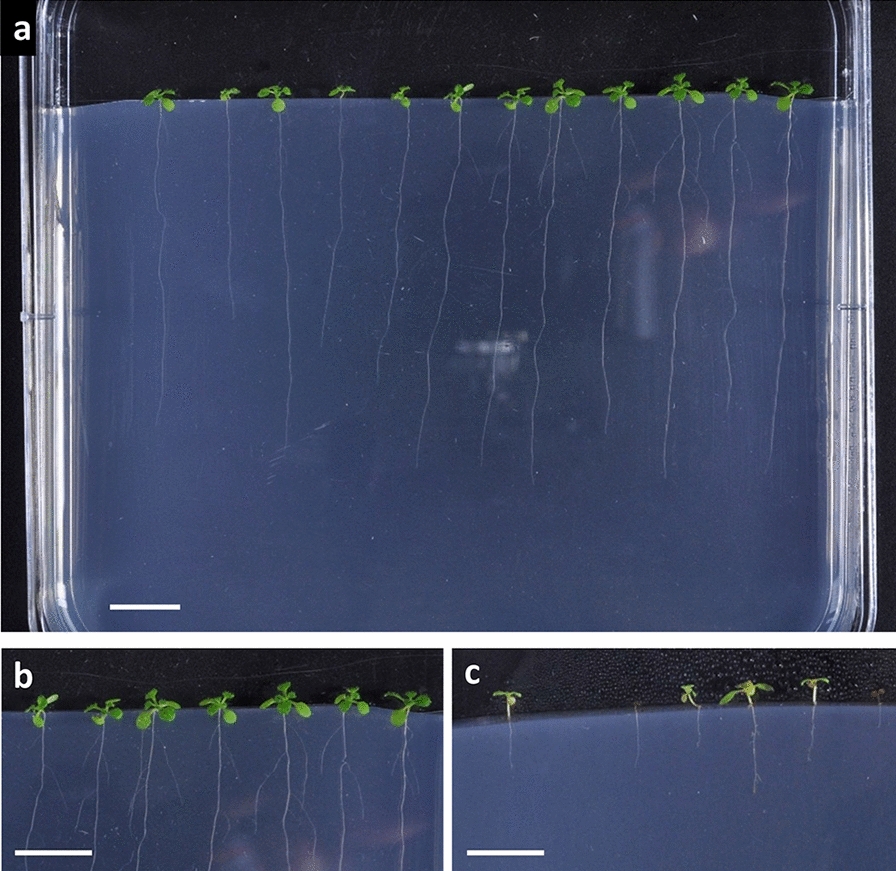
Pictures of 14 days old Col-0 seedlings in vertical square plates filled with MS modified agar medium supplemented with 5 mM KNO_3_ (scale bars = 1 cm). **a** Overview of a square plate. **b** Seedlings treated with sterile water. **c** Seedlings inoculated with *A. brassicicola*

A new monitoring system, allowing an extensive study of the effects of the fungus on seedling growth, was developed (Fig. 1). Seeds of the *A. thaliana* Columbia (Col-0) ecotype, either treated with sterile water (H_2_O) or inoculated with the Abra43 fungus, were sown in vertical square plates on agar Murashige and Skoog [37] modified medium supplemented with 5 mM NO_3_^−^ (see the Methods section; Fig. 1a). Using this system, seedlings can develop at least to the four-leaf stage, and the impact of the fungus on both seedling aerial and root system development can be monitored, as shown in the Fig. 1b and c with four-leaf stage seedlings treated with H_2_O and inoculated seedlings affected by the fungus, respectively.

#### Choice of seed inoculation method

**Fig. 2:**
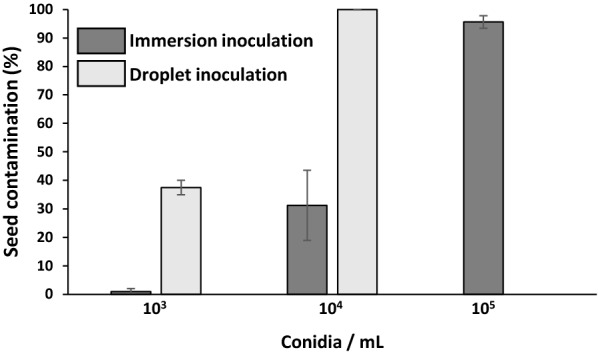
Seed contamination percentage observed 3 DAI for the immersion and droplet methods at different *A. brassicicola* conidial suspension concentrations (10^3^, 10^4^ and 10^5^ conidia per mL). Values are means of 20 seeds per biological replicate, 8 biological replicates. Error bars indicate standard error of the mean (SEM)

**Fig. 3:**
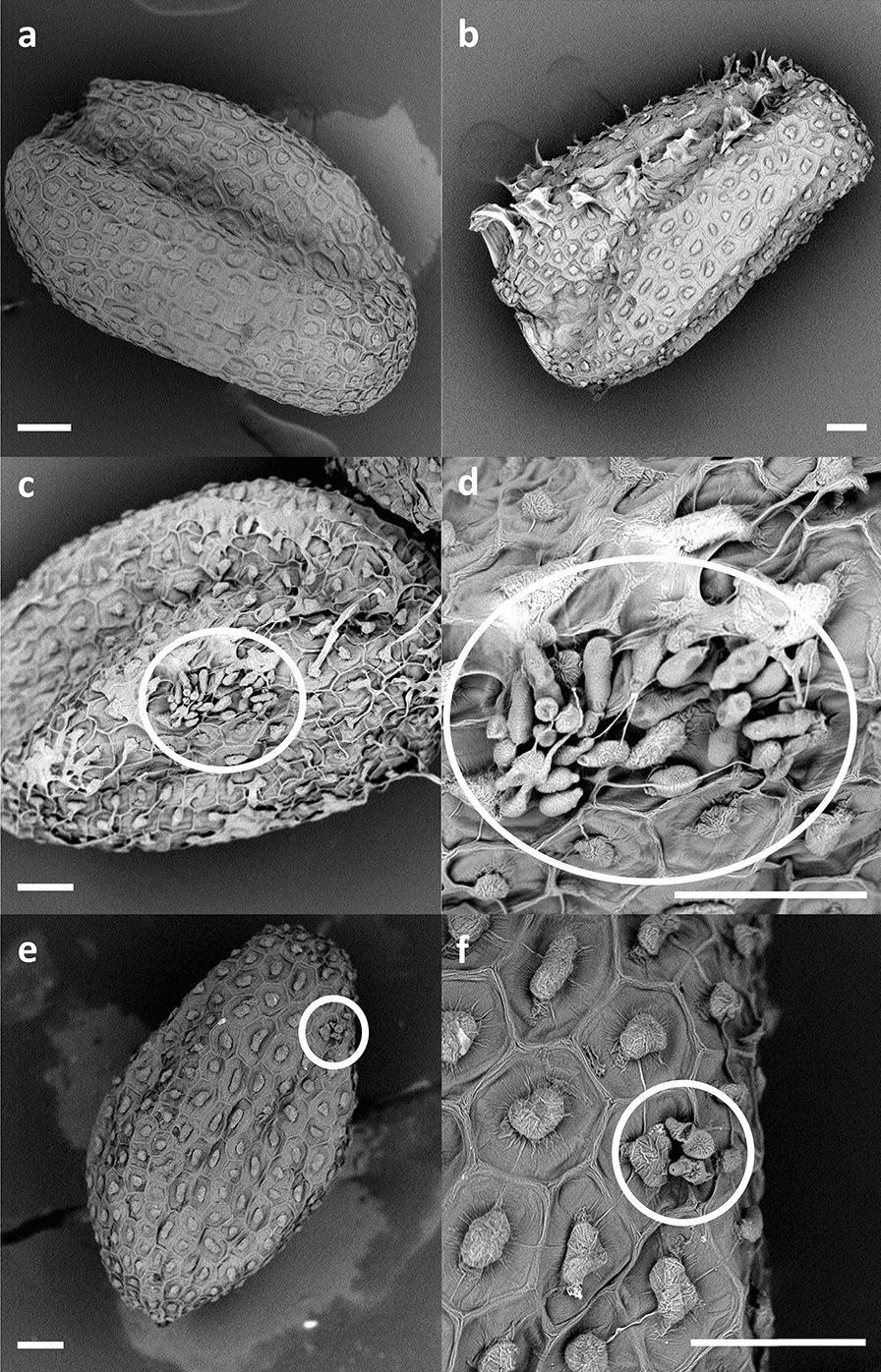
Scanning electron microscopy observations showing the heterogeneity of *A. thaliana* seed contamination with the immersion method compared to the droplet method, white circles highlighting *A. brassicicola* conidia. The white scale bars show 50 μm. **a** Seed treated with sterile water. **b** Seed inoculated with the immersion method (10^5^ conidia. mL^−1^) showing no *A. brassicicola* conidia on its surface. **c** Seed inoculated with the immersion method (10^5^ conidia. mL^−1^) presenting a cluster of conidia on its surface, **d** zoom on the conidia cluster. **e** Seed inoculated with the droplet method (10^4^ conidia. mL^−1^) presenting 2 conidia on its surface, **f** zoom on the conidia

Two methods were tested for seed inoculation with *A. brassicicola*: (i) seed immersion in a conidial suspension (10^3^, 10^4^ or 10^5^ conidia per mL) and (ii) deposition of a droplet of conidial suspension (10^3^ or 10^4^ conidia per mL) on each individual seed. Results obtained at 3 days after inoculation (DAI) showed that the droplet method of inoculation allowed 100% of the seeds to be contaminated using a suspension of 10^4^ conidia per mL contrary to the immersion method which allowed a lower and heterogeneous contamination rate, even with 10^5^ conidia per mL (Fig. 2). In addition, even though the contamination rate is close to 100% with the immersion method of inoculation at 10^5^ conidia per mL, observations of 50 seeds by scanning electron microscopy revealed that conidia were not homogeneously distributed on the seed surface, with three categories of conidia distribution (Fig. 3), seeds exhibiting either no conidia (Fig. 3b), a few conidia (not illustrated) or a cluster of conidia (Fig. 3c and d) on their surface. It is also important to note that symptoms on seedlings were too severe with 10^5^ conidia per mL preventing the observation of seedlings at 14 DAI (data not shown), which could be explained by the conidial clusters, as illustrated on Figs. 3c and d. On the contrary, none of the 210 observed seeds inoculated with the droplet method with 10^4^ conidia per mL presented any cluster of conidia on their surface, but only a few scattered conidia (Fig. 3e and f). The droplet method of inoculation with a suspension of 10^4^ conidia per mL was thus chosen as it allowed a more homogeneous seed contamination, as well as a compromise between disease severity and seedling development.

#### Development of an image analysis method to monitor the impact of the fungus on seedling aerial development

**Fig. 4:**
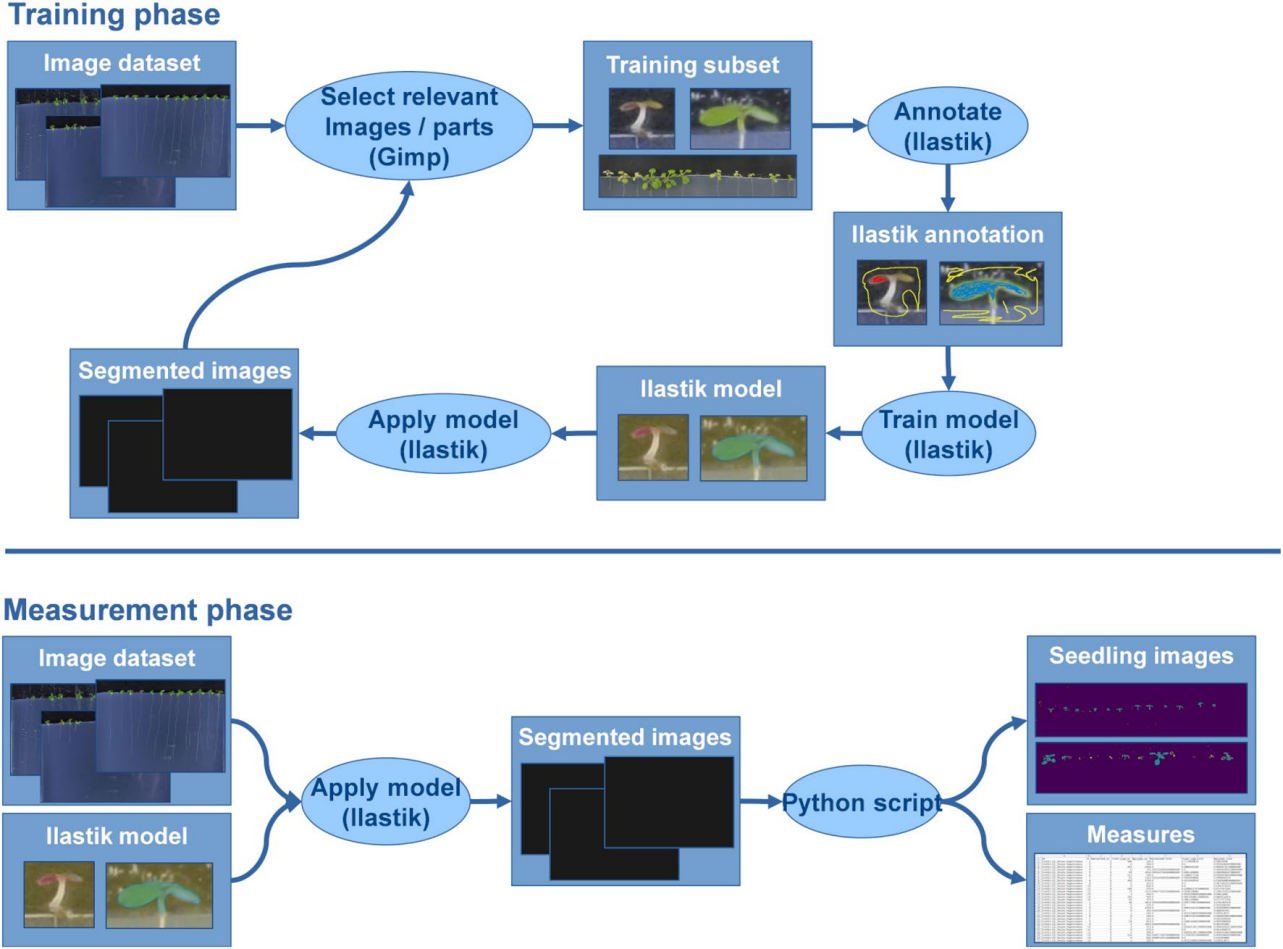
Overview of the semi-automated pipeline used to detect and calculate the green and necrotic areas per square plate. The pipeline is divided in two steps: **a** a model training phase on a sample of images and **b** the analysis of every image and computation of the surfaces. In the training phase, blue pixels correspond to green areas, red pixels to necrotic areas and yellow pixels to the background

In order to evaluate the impact of the N supply condition, of the fungus and of their interaction on seedling aerial part growth, a method based on a semi-automated image analysis pipeline was developed associating the Ilastik software [38] with a Python script. The first stage of the process consisted in training a classification random forest model with Ilastik. Twenty-five representative images were selected and annotated by associating pixels to a class. Three classes were defined corresponding to healthy tissues (green), necrotic tissues (brown) and all other objects present in the image that do not correspond to aerial parts of seedlings (Fig. 4a). Once the model trained, it was applied to the other images to identify the healthy (green) and necrotic (brown) pixels of aerial parts of the seedling (leaves, cotyledons and hypocotyl) per square plate (Fig. 4b). Classification results were then subjected to the Python script to evaluate the areas of healthy and necrotic tissues and generate colour-coded final images. The model was validated on a technical standpoint by comparing the spatial overlap between 30 representative seedlings annotated by the Ilastik model or by an expert observer using the ImageJ software, as reported in the Methods section.

To express the results, a healthy area index (HAI) representing the impact of the fungus on seedling aerial growth was developed. It depicts the percentage of healthy area variation between seedlings treated with H_2_O (${\text{HA}}_{{{\text{H}}_{2} {\text{O}}}}$) and Abra43 (HA_Abra43_) and integrates both direct and indirect fungal effects (area deficit due to necrotic tissues and seedling growth deficit, respectively).

$${\text{HAI}}\,{ = }\,{{(({\text{HA}}_{{{\text{H}}_{2} {\text{O}}}} - {\text{HA}}_{{{\text{Abra43}}}} } \mathord{\left/ {\vphantom {{(({\text{HA}}_{{{\text{H}}_{2} {\text{O}}}} - {\text{HA}}_{{{\text{Abra43}}}} } {{\text{HA}}_{{{\text{H}}_{2} {\text{O}}}} )*100}}} \right. \kern-\nulldelimiterspace} {{\text{HA}}_{{{\text{H}}_{2} {\text{O}}}} )*100}}$$

The impact on the fungus was also measured using a visual rating scale (VRS) to quantify the symptom severity on the aerial parts of seedlings. The VRS integrated both colonisation by the mycelium and necrotic area, evaluated on seedlings, and was based on a score ranging from 0 (no symptom) to 5 (100% of surface area colonised and dead seedlings) (Table 1) assigned by an expert observer.

**Table 1:** Illustration and description of the visual rating scale (VRS) used to evaluate the symptoms observed on the aerial parts of the seedlings

Illustration of a representative cotyledon/seedling	Visual rating description
	Rating = 0 No mycelium colonization nor necrosis on seedlings No impact on seedling development, four-leaf stage seedlings in general
	Rating = 1 Minimal mycelium colonization and necrosis on seedlings (< 25% of surface area) No impact on seedling development, four-leaf stage seedlings in general
	Rating = 2 Mycelium colonization and necrosis on seedlings (25% < necrosis < 50% of surface area) Small impact on seedling development, smaller four-leaf stage seedlings in general
	Rating = 3 Important mycelium colonization and necrosis on seedlings (50% < necrosis < 75% of surface area) Impact on seedling development, two-leaf stage seedlings in general
	Rating = 4 Almost total mycelium colonization and necrosis on seedlings (75% < necrosis < 99% of surface area) Important impact on seedling development, cotyledon stage seedlings in general
	Rating = 5 Total mycelium colonization and necrosis on seedlings (= 100% of surface area) Dead cotyledon stage seedlings

The scale integrated colonization by the mycelium and necrotic area on cotyledon and hypocotyl criteria, and was based on a score ranging from 0 to 5 (no symptoms to 100%) assigned by an expert observer

**Fig. 5:**
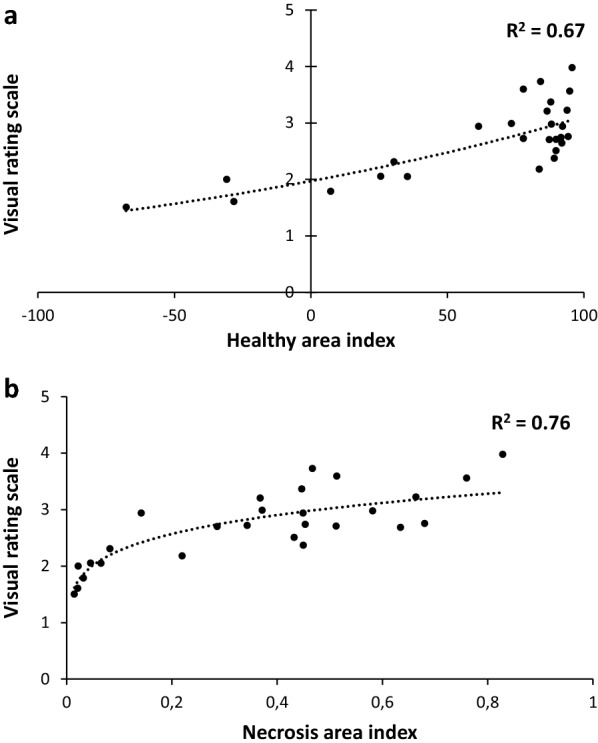
Correlation between the Ilastik-based indexes and the visual rating scale (VRS). Each point corresponds to the mean of three plates of 12 seedlings for a set of conditions. **a** VRS as a function of healthy area index, supplemented with the estimated correlation curve and R^2^ coefficient. **b** VRS as a function of necrosis area index, supplemented with the estimated correlation curve and R^2^ coefficient

In order to find out if the HAI and the VRS produced similar results regarding the fungal effect on seedlings, the correlation between the two methods was investigated (Fig. 5a). If the results of two methods shared a similar tendency, they did not correlate very well (R^2^ = 0.67, exponential function, y = 1.9709e^0.0046^), with a very uneven distribution. Another way to evaluate the fungal impact on seedlings, closer to what is measured with the VRS, would be the necrosis area index (NAI) obtained by dividing the necrosis area by the total area (necrosis + healthy area) of a squared plate. This index correlated better with the VRS (R^2^ = 0.76, power function, y = 3.4141x^0.1764^; Fig. 5b) and presented a very balanced distribution, indicating that it seemed possible to infer the results of VRS from the measures evaluated with the Ilastik model.

It is to note that both HAI and NAI appeared to assess the fungal impact in a more satisfactory way than the VRS. Firstly, VRS being based on a rating given by an observer makes it more qualitative and less objective than methods based on pixel quantification by a software. Secondly, the comparison of the two most correlated methods showed that NAI distribution of values evenly covered the full range of possible values (from 0 to 1) while VRS was only restricted to the 1.5–4 range, the two extremes of the scale being totally unused and not allowing for an adequate representation and discrimination of the fungal impact high variability. Finally, HAI was deemed superior to NAI because it was a more complete representation of the fungal effect on seedlings. Indeed, NAI measured solely the necrosis areas while HAI considered not only the necrosis areas, which are deduced of the measured healthy areas, but also the healthy area deficit caused by the fungal presence, dead seedlings and non-developed organs mostly which are very important aspects of the fungal impact on seedlings. HAI was thus chosen to express the impact of the fungus on seedling growth.

### The new monitoring system reveals different and specific seedling susceptibility to the fungus depending on nitrogen conditions at the seedling stage

The monitoring system was developed using the Col-0 ecotype on a 5 mM of NO_3_^−^ agar medium with seeds treated with either sterilised water or conidia suspension. To confirm whether it enables discriminating susceptibility to the fungus between genotypes and N growth conditions, three ecotypes presenting different susceptibility to the pathogen at adult stage [39] were tested on different media varying in concentration and form of N. The selected ecotypes were Col-0 (resistant), Wassilewskija (Ws; susceptible) and Landsberg *erecta* (L*er*; susceptible) which were grown on media in presence of 5 mM of NO_3_^−^, 0.1 mM of NO_3_^−^ or 5 mM of NH_4_^+^.

#### Seedling aerial growth is affected by the fungus depending on genotype and nitrogen conditions

**Fig. 6:**
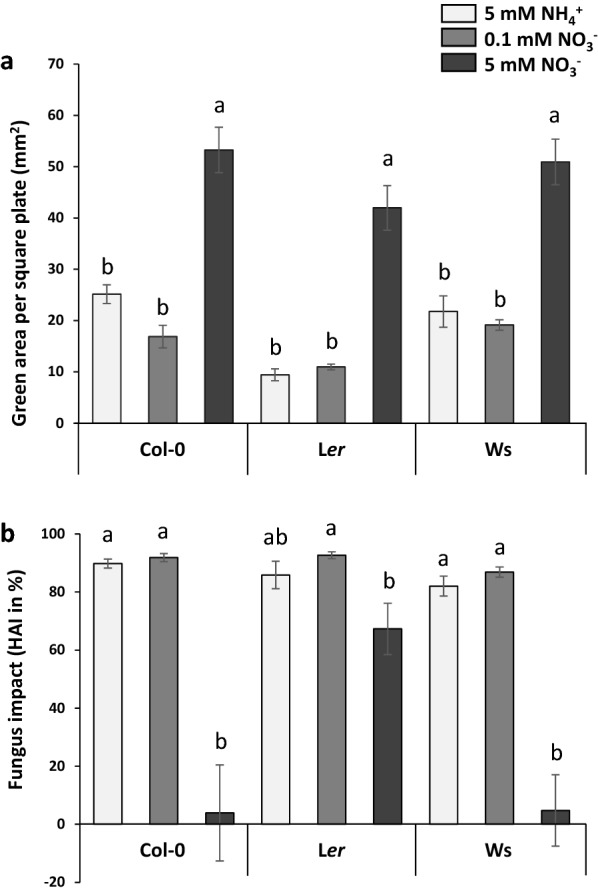
Aerial part development for Col-0, L*er* or Ws seedlings grown on different nutritive media (5 mM NH_4_^+^, 0.1 mM NO_3_^−^ or 5 mM NO_3_^−^) at 14 DAI. **(a)** Green area per square plate in the absence of the fungus (seeds inoculated with sterile H_2_O). **(b)** Green area ratio between seedlings treated with H_2_O and seedlings inoculated with Abra43 (HAI). Twelve seedlings per square plate, 3 square plates per independent experiment, 3 independent experiments. Error bars indicate SEM. Letters denote significant differences at the 0.05 level between N conditions, inside each genotype

The developed image analysis pipeline (Fig. 4) has allowed quantifying the aerial growth of seedlings from seeds treated with H_2_O by measuring their green area (${\text{HA}}_{{{\text{H}}_{{2}} {\text{O}}}}$; Fig. 6a; Additional file 1: Table S1a). In absence of the fungus, seedling aerial green area was higher under 5 mM NO_3_^−^ nutrition, with an average of 48.7 mm^2^ per square plate, compared to under the two others N media (18.8 and 15.7 mm^2^ in average for 5 mM NH_4_^+^ and 0.1 mM NO_3_^−^, respectively) (Fig. 6a; Additional file 1: Table S1a). Significant differences were also found between genotypes, with L*er* showing lower green leaf area than Col-0 and Ws under both 5 mM NH_4_^+^ and 0.1 mM NO_3_^−^ (Additional file 1: Table S1a). Then, using the calculated HAI allowed revealing the effect of N media on susceptibility to the fungus (Fig. 6b; Additional file 1: Table S1b). Seedlings grown under 5 mM NH_4_^+^ or 0.1 mM NO_3_^−^ were dramatically affected, with a fungal impact amounting for more than 80% of green area deficit for both N conditions and for all genotypes, compared to the H_2_O treated seedlings (Fig. 6b; Additional file 1: Table S1b). On the contrary, seedlings grown under 5 mM NO_3_^−^ were less susceptible to the fungus (green area reduced by 21% on average). It is also important to note the significant interaction between genotype and N nutrition for HAI (*P* < 0.0001; Additional file 1: Table S1b), notably explained by the different response of genotypes under 5 mM NO_3_^−^, with both Col-0 and Ws seemingly unaffected in their growth (− 4% and + 5% of fungal impact on green area, respectively), and by contrast, L*er* that was highly impacted (67% reduction of green area). In addition to the different susceptibilities to the fungus revealed depending on N media and genotypes, infected seedlings grown under 5 mM NO_3_^−^ appeared particularly healthy as they were at the same time more developed in control conditions and less affected by the fungus. NAI data corroborate the global conclusions obtained with the HAI (seedlings less susceptible under 5 mM NO_3_^−^ nutrition). They are presented in Additional file 2: Table S2.

#### The presence of fungus also impacts primary root elongation depending on genotype and nitrogen conditions

**Fig. 7:**
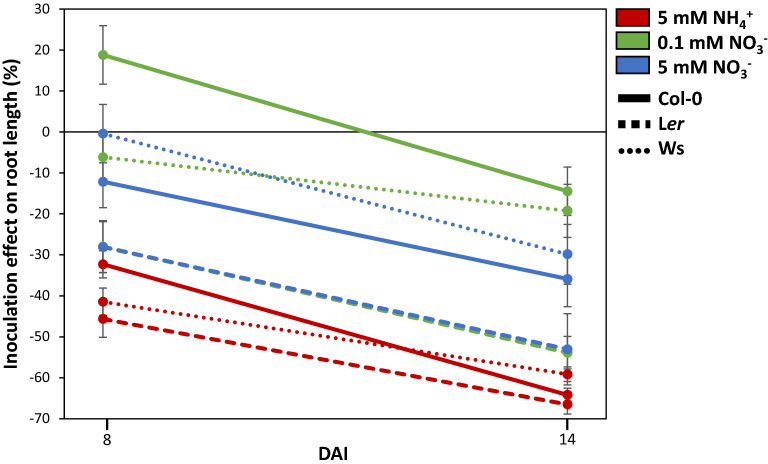
Primary root length (PRL) variation between H_2_O and Abra43 seedlings at 8 and 14 DAI, expressed as a percentage and calculated as follows: 100 * [(PRL_Abra43_−PRL_H2O_)/PRL_H2O_]. Red lines correspond to 5 mM NH_4_^+^, green lines to 0.1 mM NO_3_^−^ and blue lines to 5 mM NO_3_^−^. Solid lines correspond to Col-0, dashed lines to L*er* and dotted lines to Ws. Error bars indicate SEM

Primary root length was measured at both 8 and 14 DAI by image analysis with the Image J software (Additional file 3: Table S3a and b; Fig. 7). At 8 DAI, root length highly differed between genotypes (G effect), N media (N effect) as well as inoculation modalities (I effect). All interactions between these factors were also significant, with the G × N effect the most significant (Additional file 3: Table S3a). L*er* presented the shortest roots compared to Col-0 and Ws. Regarding N effect, roots were the shortest under 5 mM NH_4_^+^ (7 mm in average) and the longest under 5 mM NO_3_^−^ (13.8 mm in average). Finally, when considering all the data, the presence of the fungus negatively affected root elongation (− 12.8% in average). This was mainly due to the NH_4_^+^ condition where the reduction was the strongest for all the genotypes (− 38.2% in average). For the NO_3_^−^ conditions, various genotype behaviours were observed in accordance with the significant interactions revealed (Additional file 3: Table S3a), and this is more visible when results were expressed as an index of response of root length to the presence *vs.* the absence of the fungus (Fig. 7). Indeed, root length was highly affected under both nitrate conditions for L*er*, but not affected for Ws. Regarding Col-0, root length decreased in the presence of the fungus under 5 mM but increased under 0.1 mM NO_3_^−^. This result suggested that, in some nutritive conditions, the presence of the fungus could have no effect or even enhance root elongation early after inoculation (Fig. 7).

After 14 days, the same main effects were found (G, N and I effects; Additional file 3: Table S3b). L*er* was still the genotype presenting the shortest roots (16.2 mm compared to Col-0 and Ws, 22.1 and 24.4, respectively). In addition, roots were still globally the shortest under 5 mM NH_4_^+^ (10.5 mm) and the longest under 5 mM NO_3_^−^ (32.4 mm), with the differences between media being amplified compared to 8 DAI (+ 50% growth for 5 mM NH_4_^+^, + 65% for 0.1 mM NO_3_^−^ and + 135% for 5 mM NO_3_^−^). Results also indicated that root elongation was highly affected by the fungus between 8 and 14 DAI (− 38.3% at 14 DAI on average for all genotypes and N conditions compared to − 12.8% after 8 DAI). This was more or less marked depending on the N medium (Fig. 7), the impact being stronger for seedlings grown on NH_4_^+^ (− 62%), than for those grown on NO_3_^−^ (− 38% and − 26% for 5 mM and 0.1 mM, respectively). Considering the combined effects, only the G × N and N × I interactions were still significant, while the G × I and G × N × I interactions were not significant anymore, which could be explained by the amplified main effect flattening the subtler differences.

#### The new monitoring system reveals a stage specific effect of nitrogen condition on susceptibility to the fungus on seedlings, compared to adult plants

**Fig. 8:**
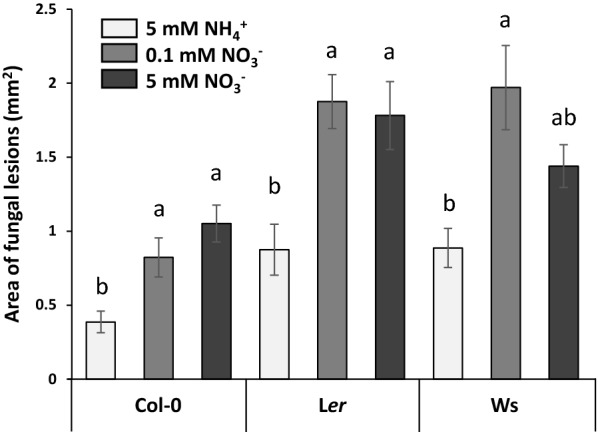
Fungal lesions for Col-0, L*er* or Ws adult plants grown on different nutritive media (5 mM NH_4_^+^, 0.1 mM NO_3_^−^ or 5 mM NO_3_^−^) in a hydroponic system, at 7 DAI. Four leaves per plant, 6 plants per independent experiment, 3 independent experiments. Error bars indicate SEM. Letters denote significant differences at the 0.05 level between N conditions, inside each genotype

To go further, findings using the developed monitoring system at the seedling stage were compared to those observed on adult plants (rosette stage) grown on a hydroponic system with the three studied N conditions (5 mM NH_4_^+^, 0.1 mM NO_3_^−^ or 5 mM NO_3_^−^). Leaf necrosis area was determined at 7 DAI. Significant differences between genotypes and between N conditions were revealed (Fig. 8; Additional file 4: Table S4). The genotype Col-0 was on average significantly less susceptible than L*er* and Ws genotypes (mean necrosis area, 0.76 mm^2^, 1.51 mm^2^ and 1.43 mm^2^, respectively) under almost all N conditions (except for the comparison with Ws under 5 mM NO_3_^−^). This difference of susceptibility to the pathogen between Col-0 and L*er* was also found at the seedling stage, whereas there was no difference at the seedling stage between Col-0 and Ws.

When comparing N conditions, plants grown on NH_4_^+^ were significantly less susceptible than those grown on NO_3_^−^ (mean necrosis area, 0.71 mm^2^, 1.51 mm^2^, and 1.42 mm^2^ for 5 mM NH_4_^+^, 0.1 mM NO_3_^−^ and 5 mM NO_3_^−^, respectively). This difference was also found when considering the genotypes separately, except for Ws under 5 mM NO_3_^−^ condition. This result contrasted very strongly with that obtained at the seedling stage, where seedlings grown under 5 mM NH_4_^+^ and 0.1 mM NO_3_^−^ were much more susceptible than those grown under 5 mM NO_3_^−^.

## Discussion

### A new monitoring system to study plant x pathogen interaction at the seedling stage

A new system allowing the monitoring of seedling growth under modulating N supply as well as the impact of the necrotrophic fungus *A. brassicicola* was developed. It permitted to follow the kinetics of both aerial and root part growth of the seedling, from germination until at least the four-leaf stage, enabling the survey of seedlings at different points in time. It also allowed for easy sampling, the seedlings being well separated and in a clean environment (no soil on the roots for example). This environment, a nutrient controlled agar medium, is another advantage of the system enabling the study of varying nutrition regimes, N forms and concentrations in this work’s case. The fully sterile system suited perfectly the study of biotic interactions at the early stages of plant development, such as the plant-fungal pathogen interaction between *A. thaliana* and *A. brassicicola* featured in the present work and could be adapted for the study of other pathosystems. Inoculation of seedlings is in most studies carried out several days after sowing [17, 23–25], preventing the study of the earlier stages of the interaction. Seed immersion in a conidial suspension is often used to solve this problem [27, 28] but, in our developed monitoring system, the droplet inoculation method implemented allowed for a more homogeneous contamination (Fig. 2). The impact of *A. brassicicola* on seedling growth was determined for both primary root elongation and seedling aerial development from image analyses. For the aerial development, a specific semi-automated pipeline was developed, based on image analyses with the Ilastik software [38] and a python script (Fig. 4). An index (HAI) was successfully applied to quantify the impact of the fungus on seedling development as it allowed accounting for every aspect of fungal impact, namely seedling death, necrosis and developmental delay. HAI was deemed the best in our study but the pipeline for image analysis is flexible enough to be used for different ways of assessing the fungal effect on plants, depending on the pathosystem or the aim of the research (e.g. necrosis alone with NAI index). The use of such a method to evaluate the fungal impact on seedlings appears more integrative than what is usually done. Indeed, studies directly inoculating the seeds commonly evaluated the infection percentage [25, 26] or the germination rate [27], and not the subsequent seedling growth, while studies inoculating already grown seedlings were restricted to latter stages and evaluated the fungal growth and sporulation [17], often with a visual qualitative scale [23, 24].

### Influence of nitrogen nutrition on fungal infection at the seedling stage

Firstly, N affected aerial development in the absence of the fungus, with seedlings grown under 5 mM NO_3_^−^ being larger than those grown under 0.1 mM NO_3_^−^ and 5 mM NH_4_^+^ (Fig. 6a). This was expected, because it is well known that NO_3_^−^ is a more advantageous N source than NH_4_^+^. Exclusive NH_4_^+^ nutrition can indeed result in toxicity symptoms in plants, which can lead to important yield losses [40], notably on *A. thaliana* [41]. However, the concentration used in our study was not stressful enough to cause leaf chlorosis, a common symptom of severe NH_4_^+^ toxicity [42]. Furthermore, given the importance of N in plant growth and development, reduced seedling growth under NO_3_^−^ deprivation conditions (0.1 mM) were also expected. Some differences between genotypes were also highlighted for seedlings grown under 0.1 mM NO_3_^−^ and 5 mM NH_4_^+^, with L*er* seedlings smaller than those of Col-0 and Ws. Regarding primary root growth, the global impact of N nutrition was similar to that found on the aerial part, with the longest primary roots belonging to seedlings grown on 5 mM NO_3_^−^ and the shortest to seedlings grown on 5 mM NH_4_^+^ (Fig. 7). This is consistent with the NH_4_^+^-induced repression of *A. thaliana* primary root growth reported in literature [43]. On the other hand, NO_3_^−^ is known for stimulating primary root growth of *A. thaliana* seedlings [44], while lower NO_3_^−^ concentrations led to a reduction of fresh root weight [45]. Differences between genotypes were found again with primary roots of Col-0 and Ws generally longer than those of L*er*, especially in the two NO_3_^−^ conditions (Additional file 3: Table S3; Fig. 7).

While the effect of N on plant-pathogen interactions has been extensively studied at the adult stage [46], studies at the seedling stage are very uncommon and most appear to be on months to years-old tree seedlings [47–50], which makes our results at this stage a novelty. The closest study was on the effect of N supply on barley seedlings inoculated with *Erysiphe graminis* f. sp*. hordei* [17], but the latter inoculation date (12 days after sowing) makes it very different from the present work. Generally, the role of N on pathogenicity can depend on both N concentration and N form [14–16]. In our study, the developed monitoring system highlighted that seedlings grown under 0.1 mM NO_3_^−^ and 5 mM NH_4_^+^ conditions were more affected than those under 5 mM NO_3_^−^ 14 days after seed inoculation (Fig. 6b). Higher NO_3_^−^ concentration was thus linked with less susceptibility, as it is the case of *Botrytis cinerea* on tomato [18], while NH_4_^+^ nutrition was linked with an increase of disease severity compared to NO_3_^−^, something that was also observed in the *Solanum lycopersicum* L.—*Fusarium oxysporum* and *Nicotiana tabacum*—*Pseudomonas syringae* interactions [19, 20]. It is important to keep in mind that the effects of N concentration and form are pathosystem specific and can have an opposite effect on plant susceptibility [17, 21, 46]. High N supply commonly enhances susceptibility to biotrophs and hemibiotrophs while reducing that to necrotrophs [21, 51, 52] which is consistent with our results.

The influence of N nutrition on fungal infection was also evaluated at the rosette stage with the same ecotypes and nitrogen media as those studied at the seedling stage. We showed that plants grown on NH_4_^+^ were significantly less susceptible than those grown on NO_3_^−^ (Fig. 8), which was consistent with a previous study we conducted with one ecotype and two N media [22]. The results obtained at the rosette stage thus contrasted strongly with those obtained at the seedling stage. The monitoring system developed to study specifically seedling growth thus allowed to demonstrate that N nutrition modulates the susceptibility of *A. thaliana* to *A. brassicicola* according to the developmental stage of the plant. Susceptibility to pathogens often vary with age, generally in the sense that younger plants are more susceptible, as demonstrated in numerous studies [53–56]. This phenomenon can be linked to leaf maturity [57] and leaf rank, [55]. For example, epidermal cuticle thickness as a physical barrier reduced the incidence of disease [58], and have been shown to increase with leaf age [59] and *A. thaliana* developmental stage [60]. It should also be noted that early seedling growth relies on both exogenous N supply and endogenous N resources from seed reserve mobilisation. This specificity may also contribute to explain the difference highlighted between the two developmental stages studied. Finally, in rosettes, the genotype Col-0 was on average significantly less susceptible than L*er* and Ws, which is consistent with the literature [39]. This difference between Col-0 and L*er* was also present at the seedling stage but, in contrast, there was no difference at the seedling stage between Col-0 and Ws, showing that genotype responses to *A. brassicicola* seem dependent on developmental stage.

## Conclusions

A new system allowing the monitoring of *A. thaliana* seedling growth under modulating N supply, as well as the impact of the necrotrophic fungus *A. brassicicola*, was developed. It enables the survey of seedlings at different points in time, in varying nutrition regimes, N forms and concentrations. A specific semi-automated image analysis pipeline was developed to quantify the impact of the fungus on seedling development. It has allowed calculating an index (HAI) accounting for every aspect of fungal impact, namely seedling death, necrosis and developmental delay, as thus highlighting the influence of N nutrition on fungal infection at the seedling stage. Seedlings subjected to NH_4_^+^ and lower NO_3_^−^ conditions were more susceptible to the fungus than those subjected to higher NO_3_^−^ conditions. The assessment of fungal impact on adult plants revealed that they were significantly less susceptible when grown on NH_4_^+^, compared to NO_3_^−^, which dramatically contrasted with findings at the seedling stage. Thanks to the monitoring system developed, it has therefore been possible to highlight that N nutrition modulates *A. thaliana* susceptibility to *A. brassicicola* according to the plant developmental stage. Because primary and secondary metabolism are of critical importance to the effect of N in the plant-pathogen interaction, elucidation of the metabolic bases associated with seedling susceptibility regulated by N supply, as well as the role of endogenous N nutrients from mobilisation of seed reserves, should provide insight into the specific findings observed at the seedling stage.

## Methods

### Biological material

Three ecotypes of *A. thaliana* (Col-0, L*er* and Ws) were studied. Seeds of each genotype were collected from plants simultaneously grown for 13 weeks under long day conditions (16 h of light at 21 °C, 8 h of darkness at 19 °C) in a growth chamber (IRHS, Angers, France). The *A. brassicicola* wild-type strain Abra43 was initially isolated from *R. sativus* seeds [7], sequenced recently [61], and was routinely grown and maintained on potato dextrose agar at 24 °C.

### Experimental monitoring system developed for Arabidopsis seedling growth

To follow seedling development and fungus-induced symptoms, *A. thaliana* seedlings were grown vertically on agar nutrient medium in square plates (12 × 12 × 1.3 cm) filled with 1.2% agar modified MS medium (0.5 mM CaCl_2_, 0.5 mM MgSO_4_, 1 mM KH_2_PO_4_, 50 µM iron-EDTA and 0.5 mL/L micro-elements buffered with 0.5 g/L MES (2-(N-morpholino)ethanesulfonic acid), pH 5.7) [37], either supplemented with 5 mM NO_3_^−^ (provided as KNO_3_), 0.1 mM NO_3_^−^ or 5 mM NH_4_^+^ (provided as NH_4_Cl). Twelve seeds per plate were sown on a plane surface obtained by cutting the medium 3 cm from the top to make easier seed sowing and inoculation but also to allow the aerial part of seedlings to develop normally. Before sowing, seeds were stratified for 3 days (in order to break their residual dormancy) and then sterilised by successive immersions, 5 min in ethanol 70°, 15 min in sodium hypochlorite (2.6% active chlorine) and three times 5 min in sterile water. Once sown, seeds were individually inoculated by deposit of a 1 µL droplet of conidia suspension (10^4^ conidia per mL; Abra43 condition) or of sterile milli-Q water (H_2_O condition). The plates were then placed for 18 days in a growth chamber under long day conditions (16 h of light at 21 °C, 8 h of darkness at 19 °C). Three independent experiments were carried out, containing each 3 plates of 12 seedlings per set of conditions.

### Seed observation by scanning electron microscopy

Seeds inoculated either by immersion (1 h in a 10^5^ conidia per mL suspension or sterile milli-Q H_2_O) or by deposit of a 1 µL droplet (10^4^ conidia per mL suspension or sterile milli-Q H_2_O) were dried and observed directly by scanning electron microscopy (Phenom™ G2 Pro) in collaboration with the IMAC platform (IMAgerie Cellulaire; SFR 4207 Quasav, Angers, France).

### Image analysis

Pictures of square plates containing seedlings were taken using a Nikon D5000 digital camera, in the same conditions of lighting and distance. The classification of images parts for the seedling aerial part analyses was performed with the Ilastik software (version 1.3.3), as presented in the global pipeline (Fig. 4). The training dataset was composed of 19 pictures of square plates (3216 × 2136 px) and 6 pictures of selected seedlings (from 176 × 328 to 1230 × 332 px). The training dataset was chosen to best represent all the variations in our data: pictures of seedlings from the three genotypes grown in different N conditions and inoculated with the fungus or not, with either homogeneity or heterogeneity in the picture regarding levels of disease severity or seedling size. These images were annotated by associating pixels to a class. Three classes were defined corresponding to healthy tissues (green), necrotic tissues (brown) and all other objects present in the image that do not correspond to aerial parts of seedlings. These annotations were then used to train Ilastik random forest model classifier. The selected features were the colour/intensity, the edge and the texture and the spatial scales for the convolution 0.70, 1.00, 1.60, and 3.50 for every feature, plus 0.30 for the colour/intensity feature. Once the model trained, it was applied to the other images to identify the healthy (green) and necrotic (brown) pixels of aerial parts of the seedling (leaves, cotyledons and hypocotyl) per square plate. Classification results were then subjected to a Python 3.7 script to evaluate the areas of healthy and necrotic tissues and generate the colour-coded final images. This script was based mainly on the numpy [62], scikit-image [63] and matplotlib [64] libraries.

The pipeline was tested on 18 pictures of square plates (3216 × 2136 px) and 5 pictures of selected seedlings (from 176 × 328 to 1230 × 332 px) to assess the quality of the classification. The evaluation of the quality of the model was based on the comparison of the areas of 30 representative seedlings measured by our image analysis pipeline and a ground truth corresponding to annotations performed manually by an expert observer with the ImageJ software (version 1.53 k) [65]. The metric selected to measure the spatial overlap between those two sets of classified pixels was the classical Dice Sørensen coefficient (DSC) = 2*|*A* ∩ *B*| / |*A*| +|*B*|. The DSC then equals twice the number of elements common to both sets (|A ∩ B|) divided by the sum of the number of elements in each set (|A| +|B|). The DSC for our data was high (0.98), showing that there was no real difference between the model and the manual annotation.

To assess the impact of variability of annotation by the expert, the random forest model was trained with the same image dataset with new annotations. The variability found was 1.6% of surface on the test data set. This was found to be negligible by comparison with the biological variation inside the conditions.

Regarding the root part, ImageJ (version 1.53 k) [65] was used to determine the length of primary roots.

### Experiments at the rosette stage

Experiments at the rosette stage were carried out according to Barrit et al. [22]. Seeds of three *A. thaliana* ecotypes (Col-0, L*er* and Ws) were sown in pots filled with soil and have been grown in a growth chamber (8 h light / 16 h darkness, 21 °C). After 4 weeks, plants were transferred individually in a hydroponic system containing modified MS medium supplemented with 5 mM KNO_3_ for 2 weeks. Afterwards, plants were placed in a new hydroponic nutrient solution containing either 5 mM NH_4_^+^ (provided as NH_4_Cl) or 0.1 mM or 5 mM NO_3_^−^ (provided as KNO_3_), as sole N source for 2 weeks. Nutritive solution was replaced every week. For plant infection assays, 5 μL drops of *A. brassicicola* (Abra43) conidia suspension (10^5^ conidia per mL) were deposited on leaves from eight weeks-old plants. For control plants, 5 µL drops of H_2_O were used. Plants were then maintained under saturating humidity (100% relative humidity) for 2 days. Foliar symptoms were quantified at 7 DAI by measuring manually the lesion area surface.

### Statistical analyses

Statistical analyses were carried out with the R software (version 4.1.2). Parametric tests (ANOVA followed by Tukey’s HSD test in the case of multiple comparisons, Student’s t test in the case of additional side-by-side comparisons) were performed when the conditions of normal distribution and homogeneity of variances were met. Otherwise, nonparametric tests (ANOVA using permutation tests for multiple comparisons, or Wilcoxon-Mann–Whitney test for additional side by side comparisons) were used to test the significance of the results. Statistically significant differences were denoted by P < 0.05.

## Supplementary Information

**Additional file 1: Table S1.** (a) Average values of green area, in square millimetres (per square plate), for Col-0, L*er* or Ws seedlings grown on different nutritive media (5 mM NH_4_^+^, 0.1 mM NO_3_^-^ or 5 mM NO_3_^-^), 14 DAI with sterile H_2_O. Twelve seedlings per square plate, 3 square plates per independent experiment, 3 independent experiments. Lowercase letters indicate a statistical difference between genotypes, inside a N condition. Uppercase letters indicate a statistical difference between N conditions, inside a genotype, for each N condition separately and for their mean. No letter indicates the absence of statistical difference for the comparison. The different factor effects and their interaction are presented at the bottom of the table, with the *P*-value. Significance of *P*: 0 < *** < 0.001. (b) Average values of green area ratio between seedlings treated with H_2_O and seedlings inoculated with Abra43 (HAI), for Col-0, L*er* or Ws seedlings grown on different nutritive media (5 mM NH_4_^+^, 0.1 mM NO_3_^-^ or 5 mM NO_3_^-^), 14 DAI. Twelve seedlings per square plate, 3 square plates per independent experiment, 3 independent experiments. Lowercase letters indicate a statistical difference between genotypes, inside a N condition. Uppercase letters indicate a statistical difference between N conditions, inside a genotype, for each N condition separately and for their mean. No letter indicates the absence of statistical difference for the comparison. The different factor effects and their interaction are presented at the bottom of the table, with the *P*-value. Significance of *P*: 0 < *** < 0.001 < ** < 0.01.

**Additional file 2: Table S2.** Average values of the necrosis area index (NAI) obtained by dividing the necrosis area by the total aerial area, for Col-0, L*er* or Ws seedlings inoculated with Abra43 and grown on different nutritive media (5 mM NH_4_^+^, 0.1 mM NO_3_^-^ or 5 mM NO_3_^-^), at 14 DAI. Twelve seedlings per square plate, 3 square plates per independent experiment, 3 independent experiments. Lowercase letters indicate a statistical difference between genotypes, inside a N condition. Uppercase letters indicate a statistical difference between N conditions, inside a genotype, for each N condition separately and for their mean. No letter indicates the absence of statistical difference for the comparison. The different factor effects and their interaction are presented at the bottom of the table, with the *P*-value. Significance of *P*: 0 < *** < 0.001.

**Additional file 3: Table S3.** (a) Average values of primary root length 8 DAI, in centimetres, for Col-0, L*er* or Ws seedlings after H_2_O treatment or Abra43 inoculation, grown on different nutritive media (5 mM NH_4_^+^, 0.1 mM NO_3_^-^ or 5 mM NO_3_^-^). Twelve seedlings per square plate, 3 square plates per independent experiment, 3 independent experiments. Lowercase letters indicate a statistical difference between genotypes, inside a condition of N and inoculation, for each genotype separately and for their mean. Uppercase letters indicate a statistical difference between N conditions, for a genotype and an inoculation condition, for each N condition separately and for their mean. Greek letters indicate a statistical difference between inoculation conditions, for a genotype and a N condition, for each inoculation condition separately and for their mean. No letter indicates the absence of statistical difference for the comparison. The different factor effects and their interaction are presented at the bottom of the table, with the *P*-value. Significance of *P*: 0 < *** < 0.001 < ** < 0.01 < * < 0.05. (b) Average values of primary root length 14 DAI, in centimetres, for Col-0, L*er* or Ws seedlings after H_2_O treatment or Abra43 inoculation, grown on different nutritive media (5 mM NH_4_^+^, 0.1 mM NO_3_^-^ or 5 mM NO_3_^-^). Twelve seedlings per square plate, 3 square plates per independent experiment, 3 independent experiments. Lowercase letters indicate a statistical difference between genotypes, inside a condition of N and inoculation, for each genotype separately and for their mean. Uppercase letters indicate a statistical difference between N conditions, for a genotype and an inoculation condition, for each N condition separately and for their mean. Greek letters indicate a statistical difference between inoculation conditions, for a genotype and a N condition, for each inoculation condition separately and for their mean. No letter indicates the absence of statistical difference for the comparison. The different factor effects and their interaction are presented at the bottom of the table, with the *P*-value. Significance of *P*: 0 < *** < 0.001.

**Additional file 4: Table S4.** Average values of fungal lesions (lesion area) for Col-0, L*er* or Ws adult plants grown on different nutritive media (5 mM NH_4_^+^, 0.1 mM NO_3_^-^ or 5 mM NO_3_^-^) in a hydroponic system, 7 DAI. Four leaves per plant, 6 plants per independent experiment, 3 independent experiments. Lowercase letters indicate a statistical difference between genotypes, inside a condition of N, for each genotype separately and for their mean. Uppercase letters indicate a statistical difference between N conditions, inside a genotype, for each N condition separately and for their mean. No letter indicates the absence of statistical difference for the comparison. The different factor effects and their interaction are presented at the bottom of the table, with the *P*-value. Significance of *P*: 0 < *** < 0.001.

## Data Availability

All data generated or analysed during this study are included in this published article and its supplementary information file. Script source code used during the current study for image analysis is available from the corresponding author on reasonable request.

## Abbreviations

DAI: Day after inoculation
DSC: Dice Sørensen coefficient
GABA: Gamma-aminobutyric acid
HAI: Healthy area index
N: Nitrogen
NAI: Necrosis area index
PRL: Primary root length
SEM: Standard error of the mean
VRS: Visual rating scale
