# Supplementary material for: A new in vitro monitoring system reveals a specific influence of Arabidopsis nitrogen nutrition on its susceptibility to Alternaria brassicicola at the seedling stage
Source: Plant Methods. 2022 Dec 8;18:131. doi: 10.1186/s13007-022-00962-3 (PMC9733346; doi:10.1186/s13007-022-00962-3)
Supplement: Supplementary file 1 — Additional file 1: Table S1. (a) Average values of green area, in square millimetres (per square plate), for Col-0, Ler or Ws seedlings grown on different nutritive media (5 mM NH4+, 0.1 mM NO3- or 5 mM NO3-), 14 DAI with sterile H2O. Twelve seedlings per square plate, 3 square plates per independent experiment, 3 independent experiments. Lowercase letters indicate a statistical difference between genotypes, inside a N condition. Uppercase letters indicate a statistical difference between N conditions, inside a genotype, for each N condition separately and for their mean. No letter indicates the absence of statistical difference for the comparison. The different factor effects and their interaction are presented at the bottom of the table, with the P-value. Significance of P: 0 < *** < 0.001. (b) Average values of green area ratio between seedlings treated with H2O and seedlings inoculated with Abra43 (HAI), for Col-0, Ler or Ws seedlings grown on different nutritive media (5 mM NH4+, 0.1 mM NO3- or 5 mM NO3-), 14 DAI. Twelve seedlings per square plate, 3 square plates per independent experiment, 3 independent experiments. Lowercase letters indicate a statistical difference between genotypes, inside a N condition. Uppercase letters indicate a statistical difference between N conditions, inside a genotype, for each N condition separately and for their mean. No letter indicates the absence of statistical difference for the comparison. The different factor effects and their interaction are presented at the bottom of the table, with the P-value. Significance of P: 0 < *** < 0.001 < ** < 0.01. [file 13007_2022_962_MOESM1_ESM.docx]

**Table S1**

**a**

**b**
