# Supplementary material for: A new in vitro monitoring system reveals a specific influence of Arabidopsis nitrogen nutrition on its susceptibility to Alternaria brassicicola at the seedling stage
Source: Plant Methods. 2022 Dec 8;18:131. doi: 10.1186/s13007-022-00962-3 (PMC9733346; doi:10.1186/s13007-022-00962-3)
Supplement: Supplementary file 3 — Additional file 3: Table S3. (a) Average values of primary root length 8 DAI, in centimetres, for Col-0, Ler or Ws seedlings after H2O treatment or Abra43 inoculation, grown on different nutritive media (5 mM NH4+, 0.1 mM NO3- or 5 mM NO3-). Twelve seedlings per square plate, 3 square plates per independent experiment, 3 independent experiments. Lowercase letters indicate a statistical difference between genotypes, inside a condition of N and inoculation, for each genotype separately and for their mean. Uppercase letters indicate a statistical difference between N conditions, for a genotype and an inoculation condition, for each N condition separately and for their mean. Greek letters indicate a statistical difference between inoculation conditions, for a genotype and a N condition, for each inoculation condition separately and for their mean. No letter indicates the absence of statistical difference for the comparison. The different factor effects and their interaction are presented at the bottom of the table, with the P-value. Significance of P: 0 < *** < 0.001 < ** < 0.01 < * < 0.05. (b) Average values of primary root length 14 DAI, in centimetres, for Col-0, Ler or Ws seedlings after H2O treatment or Abra43 inoculation, grown on different nutritive media (5 mM NH4+, 0.1 mM NO3- or 5 mM NO3-). Twelve seedlings per square plate, 3 square plates per independent experiment, 3 independent experiments. Lowercase letters indicate a statistical difference between genotypes, inside a condition of N and inoculation, for each genotype separately and for their mean. Uppercase letters indicate a statistical difference between N conditions, for a genotype and an inoculation condition, for each N condition separately and for their mean. Greek letters indicate a statistical difference between inoculation conditions, for a genotype and a N condition, for each inoculation condition separately and for their mean. No letter indicates the absence of statistical difference for the co [file 13007_2022_962_MOESM3_ESM.docx]

**Table S3**

**a**

**b**
